# A clinico-radiological evidence of Ayurvedic management for presumed inflammatory/idiopathic pleural effusion: case report

**DOI:** 10.3389/fmed.2026.1803298

**Published:** 2026-07-14

**Authors:** Amit Nakanekar, Yogita Pole

**Affiliations:** Department of Kayachikitsa, Government Ayurved College and Hospital, Nagpur, India

**Keywords:** case report, conservative management, herbal medicine, idiopathic pleural effusion, integrative medicine, ayurveda

## Abstract

**Background:**

Pleural effusion is a common cause of respiratory morbidity and may present diagnostic and therapeutic challenges, particularly in resource-limited settings. This case report describes the clinical observations of a clinically stable patient with presumed inflammatory/idiopathic pleural effusion managed using a conservative, non-invasive Ayurvedic approach.

**Case presentation:**

We present the case of a 34-year-old man who presented with progressive left-sided pleuritic chest pain [8/10 on the visual analogue scale (VAS)], exertional dyspnoea (shortness of breath with activity, grade 2 on the modified Medical Research Council scale and with a score of 3 on the Modified Borg Dyspnoea Scale), dry cough, chest discomfort, loss of appetite (anorexia), and irritability. Chest x-ray and ultrasonography confirmed a moderate left-sided pleural effusion. Sputum for acid-fast bacilli (AFB) and for the cartridge-based nucleic acid amplification test (CBNAAT) was negative. The patient was treated with classical Ayurvedic formulations (Lakshmivilas Rasa, Lakshadi Guggulu, and herbal powder combinations) for 12 days.

**Results:**

After treatment, the patient’s dyspnoea resolved completely (mMRC Grade 0; Modified Borg Dyspnoea Scale score 0). Chest pain decreased markedly (VAS 1/10). Inflammatory markers were normalised. Follow-up chest x-ray showed near-complete radiological resolution of the pleural effusion without the need for pleural tapping.

**Conclusion:**

This case report describes clinical and radiological improvements in a patient with presumed inflammatory/idiopathic pleural effusion managed using a safe, conservative, non-invasive, and cost-effective Ayurvedic approach. Although clinical and radiological improvements were observed, further well-designed, multicentre pragmatic studies incorporating standardised diagnostic evaluation, pleural fluid analysis, inflammatory biomarkers, and long-term follow-up are required to assess the reproducibility of these observations and explore potential causal relationships.

## Introduction

Pleural effusion is common across the Indian subcontinent and has various causes. Recent studies have shown that non-tubercular pleural effusions account for 25.5–76.5% of cases, while tuberculosis was once considered the main cause. Reports have also indicated cancer (12–23%), parapneumonic effusions (10–13%), congestive heart failure (5–15%), connective tissue disorders, and idiopathic or unknown causes (3–25%) (1).

Unilateral idiopathic pleural effusion is a diagnostic and therapeutic challenge. It often requires extensive tests to rule out infection, cancer, or systemic disease. However, many cases continue to remain unexplained despite detailed evaluation (2, 3).

Thoracocentesis is the primary method for diagnosing and managing pleural effusions in modern practice. It usually provides symptom relief. However, this invasive procedure carries risks: pneumothorax, bleeding, infection, re-expansion pulmonary oedema, and recurrence, especially in simple or recurring cases. These limitations highlight the need for carefully selected conservative management strategies. These are important for patients who may not need invasive intervention (4, 5).

Ayurvedic texts describe symptoms of pleural effusion under the term *Uraḥstoya,* found in Bhaishajya Ratnavali and Madhavnidan. This condition involves excess *Ambu* (serous fluid) in the thoracic cavity *(Shleshmadhara Kala of Phupphusa)*, with symptoms similar to those of a pleural effusion.

For unilateral pleural effusions, if critical causes are ruled out and medical therapy might suffice, conservative and non-invasive therapies are valuable. These methods provide affordable care in areas with limited infrastructure, where procedures may not be available.

We report an affordable Ayurvedic approach to managing unilateral presumed inflammatory/idiopathic pleural effusion. The case showed clinical, haematological, and radiological improvement with non-invasive methods. This observation suggested a potential role for such an approach in carefully selected patients.

### Patient information

A 34-year-old man presented with a 10–15-day history of progressive left-sided pleuritic chest pain (VAS 8/10), exertional dyspnoea (mMRC Grade 2; Modified Borg Dyspnoea Scale score 3), dry cough, and chest discomfort. Associated symptoms include anorexia, irritability, and unintentional weight loss. He reported a brief episode of fever with chills 2 days prior, which resolved after short-term antipyretic therapy. No prior similar episodes or long-term medication use were reported. No definitive prior treatment for the present condition was undertaken apart from symptomatic management, and no sustained clinical improvement was observed. The patient had a history of occasional alcohol intake for 4–5 years and chronic tobacco and kharra use for approximately 10 years. There was no known history of tuberculosis, chronic respiratory disease, malignancy, or significant family history.

### Clinical findings

On general examination, the patient appeared lean-built, alert, and oriented. No icterus, pallor, clubbing, cyanosis, or lymphadenopathy was present. Vital signs revealed hypotension (BP 90/60 mmHg), mild tachycardia (pulse 101/min), and tachypnoea (28 breaths/min). Oxygen saturation was normal (SpO₂ 98% in room air). Respiratory examination revealed less chest expansion on the left side. Percussion showed dullness in the left lower lung. Auscultation demonstrated absent air entry over the left lower lung zone, with associated crepitus in the left upper and lower lobes, suggestive of unilateral pleuropulmonary involvement. Cardiovascular examination revealed normal heart sounds (S1 and S2), and other systemic examinations were unremarkable (Table 1).

**Table 1:** Clinical, functional, and radiological assessments before and after treatment.

Assessment domain	Parameter	Before treatment	After treatment
Subjective	Pleuritic chest pain (VAS)	8/10	1/10
	Dyspnoea (mMRC grade)	Grade 2	Grade 0
	Dyspnoea (modified Borg Dyspnoea grade)	Grade 3	Grade 0
	Cough	+++	Absent
	Chest discomfort	+++	Absent
	Irritability	+++	Absent
	Anorexia	++++	Absent
Objective	Respiratory rate (breaths/min)	28	18
	Blood pressure (mmHg)	90/60	110/70
	Respiratory examination	Absent air entry in the left lower zone with crepitus	Bilateral equal air entry, no added sounds
Functional scale	mMRC and ATS Score	2	0
	Modified Borg Dyspnoea grade	3	0
Radiological	Chest X-ray (PA view)	Moderate left-sided pleural effusion	Near-complete resolution of pleural effusion
Electrophysiological	ECG	within normal limits	within normal limits
Haematological	Haemoglobin (g/dL)	10.6	13.2
	Total leukocyte count (/cumm)	7,900	7,000
	Neutrophils (%)	69	67
	Lymphocytes (%)	16	24
	Eosinophils + Monocytes (%)	15	9
	ESR (mm/h)	86	18
	Platelet count (lacs/cumm)	6.79	2.74
	Haemoglobin (g/dL)	10.6	13.2
LFT	Total bilirubin (mg/dL)	0.35	0.43
	Direct bilirubin (mg/dL)	0.19	0.21
	SGOT (U/L)	45.2	21.2
	SGPT (U/L)	75.5	14.2
KFT	Blood urea (mg/dL)	10	26.9
	Serum creatinine (mg/dL)	0.80	1.0
	Uric acid (mg/dL)	4.2	5.8
Lipid profile	Cholesterol (mg/dl)	152	187
	Triglycerides (mg/dl)	115.4	112.3
	HDL (mg/dl)	36.6	45.7

### Ashtavidha pariksha (8-fold examination)

*Nadi (pulse) – 101/min, Mala (stool) – normal, Muta (urine) – normal, Jivha (tongue) – clear, Shabda (voice) – slightly weak (Swaradaurba), Sparsha (skin to touch) – warm, Druka (vision) – clear, Akruti (appearance) – thin build (BMI 15.9 kg/m^2^).*

**Figure 1:**
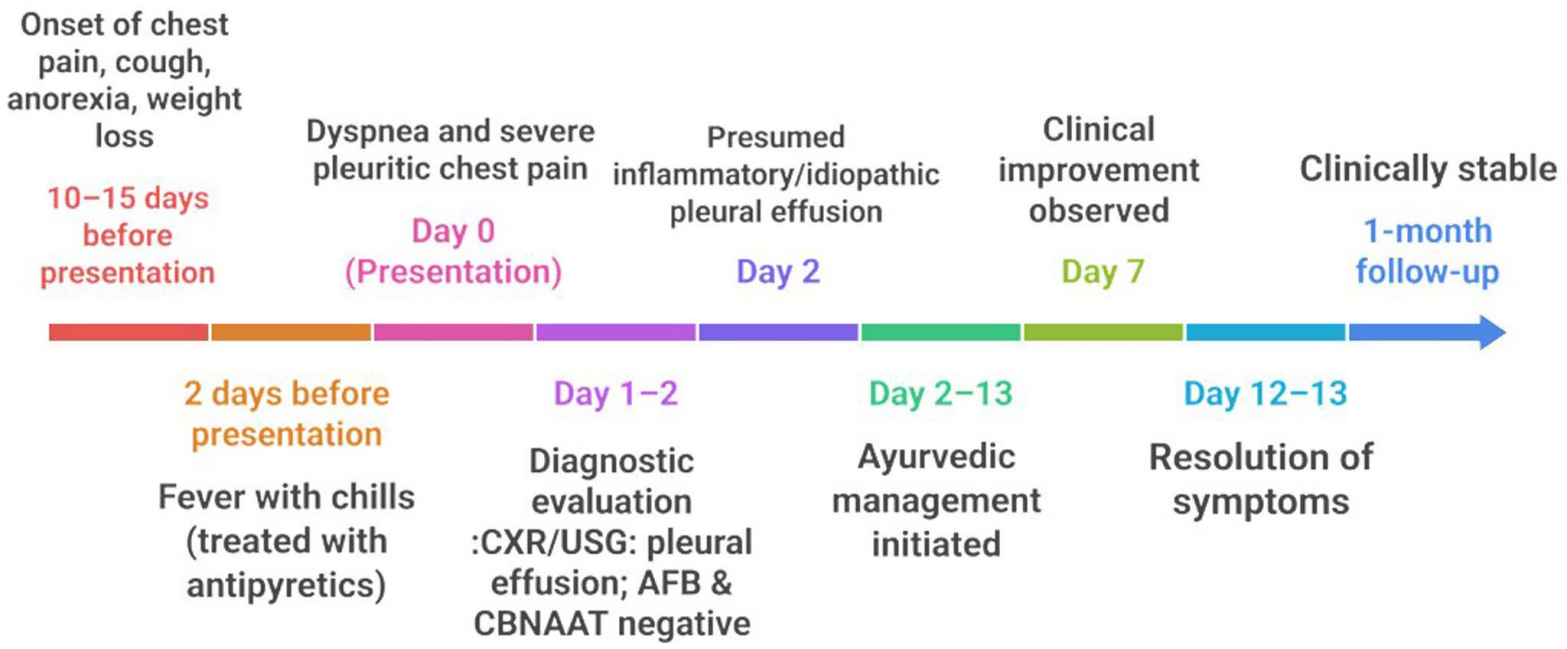
Timeline.

Timeline: mentioned in Figure 1.

### Diagnosis assessment

The diagnostic assessment was based on an integrated evaluation of clinical findings, radiological investigations, microbiological tests, and serological parameters, with key diagnostic features summarised in Table 2.

**Table 2:** Diagnosis assessment.

Domain	Parameter	Findings/interpretation
Clinical assessment	Presenting symptoms	Progressive left-sided pleuritic chest pain (VAS 8/10), exertional dyspnoea (mMRC Grade 2, Modified Borg Dyspnoea Score 3), dry cough, anorexia, weight loss, and irritability.
	Vital parameters	Blood pressure: 90/60 mmHg; pulse rate: 101/min
	Respiratory examination	Absent air entry into the left lower lung zone with crepitations
Radiological assessment [Appendix A]	Chest radiograph	Left-sided pleural effusion
	Ultrasonography	Moderate left-sided pleural effusion
Microbiological assessment [Appendix D]	Sputum AFB	Negative
	CBNAAT (Xpert MTB/RIF)	Negative
Serological assessment [Appendix. C]	HBsAg	Non-reactive
	HIV 1 and 2 antibodies	Non-reactive
Laboratory interpretation	Significance	Absence of viral hepatitis or HIV-associated immunosuppression reduces the likelihood of opportunistic infections, chronic liver disease–related effusion, and secondary tubercular or malignant pleural involvement.
Haematological assessment [Appendix B]	ESR, platelet count, and haemoglobin	Elevated ESR and reactive thrombocytosis with mild anaemia, suggestive of active systemic inflammation without evidence of sepsis or hematologic malignancy.
Etiological evaluation	Tuberculosis	Considered less likely due to the non-occurrence of symptoms and a normal chest X-ray for 1 year, but due to the absence of pleural fluid analysis, it cannot be ruled out completely.
	Viral/immunodeficiency-related causes	Considered unlikely based on negative serology
	Malignancy	Considered less likely due to the non-occurrence of symptoms and a normal chest X-ray for 1 year, but due to the absence of pleural fluid analysis, it cannot be ruled out completely.
Final diagnosis		Presumed inflammatory/idiopathic pleural effusion
Clinical severity scales	mMRC dyspnoea scale	Grade 2
	ATS dyspnoea grade	Grade 2
	Modified Borg Dyspnoea grade	Grade 3
	Visual Analogue Scale (VAS)	8/10
Ayurvedic correlation	Classical diagnosis	*Uraḥstoya*

Chest X-ray and ultrasonography confirmed the presence of moderate left-sided pleural effusion (Appendix A). Microbiological investigations, including sputum AFB smear and CBNAAT (Xpert MTB/RIF), were negative (Appendix D), thereby reducing the likelihood of active pulmonary tuberculosis. Serological tests, including HIV-1 and HIV-2 antibodies and HBsAg, were non-reactive (Appendix C), thereby reducing the probability of immunocompromised states and related secondary causes.

Haematology (Appendix B) showed high ESR, reactive thrombocytosis, and mild anaemia, indicating an underlying inflammatory process. There was no sign of sepsis or haematological malignancy.

### Diagnostic reasoning and differential diagnosis

Tubercular pleural effusion was initially suspected given the high regional prevalence; however, it was considered less likely because of a negative sputum AFB smear and CBNAAT, the absence of persistent fever, and subsequent clinical and radiological improvement without anti-tubercular therapy.

Malignant pleural effusion was considered less likely given the patient’s age, the absence of radiological evidence of a mass lesion, and favourable clinical progression. However, in the absence of pleural fluid cytology and further diagnostic evaluation, malignancy could not be definitively excluded.

Parapneumonic effusion was considered less likely. There was an absence of persistent fever, radiological consolidation, leukocytosis, or features of active infection.

Cardiac, hepatic, and renal causes (transudative effusion) were considered unlikely due to the absence of suggestive clinical features and normal ECG, liver function, and renal function parameters both before and after treatment (Table 1; Appendices B, E).

### Diagnostic challenges

Pleural fluid analysis was not performed, as thoracocentesis was deferred following an individualised clinical risk–benefit assessment and shared decision-making with the patient. At the initial evaluation, pleural tapping was explained to the patient as part of the standard diagnostic workup. However, the patient expressed reluctance and preferred to initially attempt conservative Ayurvedic management after discussion with family members.

Treatment was continued for 12 days under close monitoring. On reassessment after 12 days, a repeat chest X-ray was performed, demonstrating near-complete resolution of the effusion, thereby reducing the clinical necessity for thoracocentesis.

There were no comorbidity-related contraindications or technical limitations. In the absence of pleural fluid analysis, classification into exudative versus transudative effusion could not be performed. Accordingly, the condition has been described as a presumed inflammatory/idiopathic pleural effusion, based on clinicoradiological correlation and the exclusion of major aetiologies.

Nevertheless, the absence of pleural fluid biochemical, microbiological, and cytological evaluation remains a major diagnostic limitation. Consequently, alternative aetiologies, including tuberculous pleuritis, parapneumonic effusion, and malignant pleural effusion, cannot be definitively excluded. However, the absence of symptom recurrence and a normal chest radiograph during a 1-year follow-up make these diagnoses less likely. The observed clinical and radiological improvements are reported as temporal associations, and causal inference regarding therapeutic intervention cannot be established.

### Prognostic characteristics

The patient was clinically stable at presentation, with normal oxygen saturation and no evidence of major systemic disease, suggesting a better prognosis.

Therapeutic Interventions: Mentioned in Table 3.

**Table 3:** Therapeutic interventions:

Treatment	Route of administration	Dose	Anupana	Kala (time of administration)	Duration
Talisadi Churna + Amalaki Churna + Yashtimadhu Churna + Ashwagandha Churna	Oral	5 gm (combined herbal powder) twice daily (BD)	Koshna Jala (lukewarm water)	Muhurmuhu (repeated doses)	12 days
Lakshmivilas Rasa	Oral	1 tablet twice daily (BD)	Koshna Jala (lukewarm water)	Vyanodane (After meals)	12 days
Lakshadi Guggulu	Oral	2 tablets twice daily (BD)	Koshna Jala (lukewarm water)	Rasayana kale and Apane (administered in the morning on an empty stomach and before a meal at evening)	12 days

### Follow-up and outcomes

**Figure 2:**
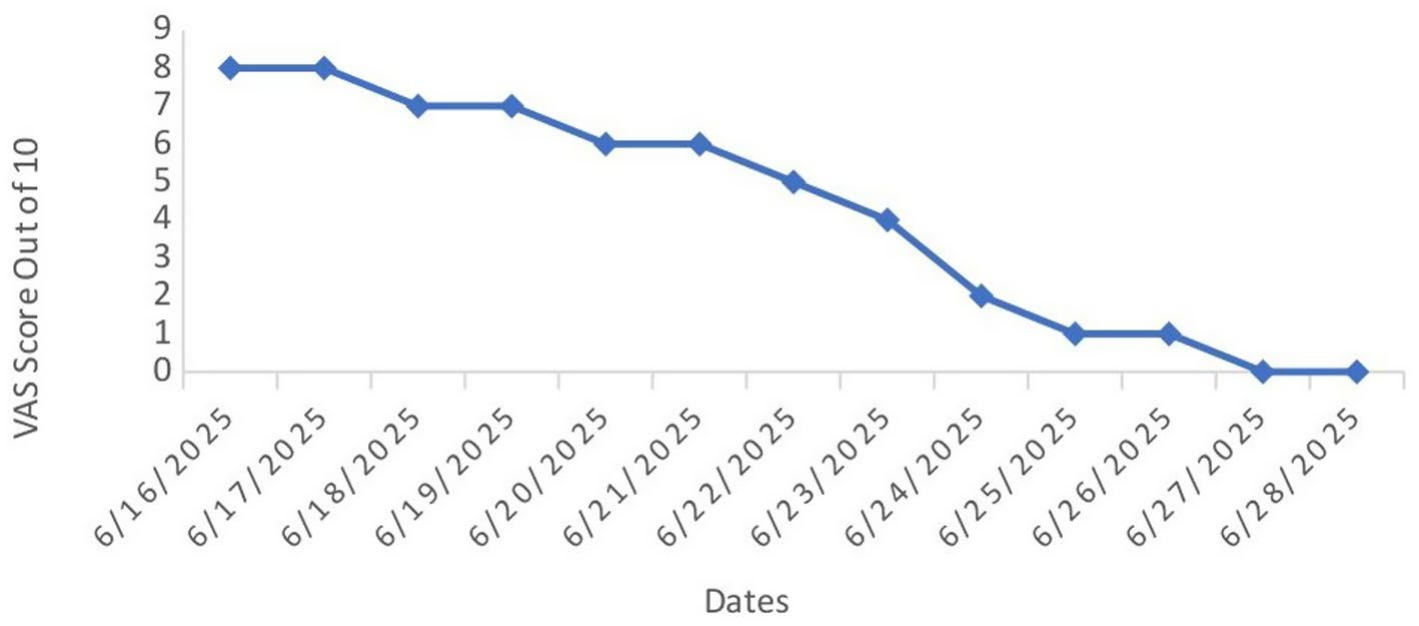
Pleuritic pain assessment.

**Figure 3:**
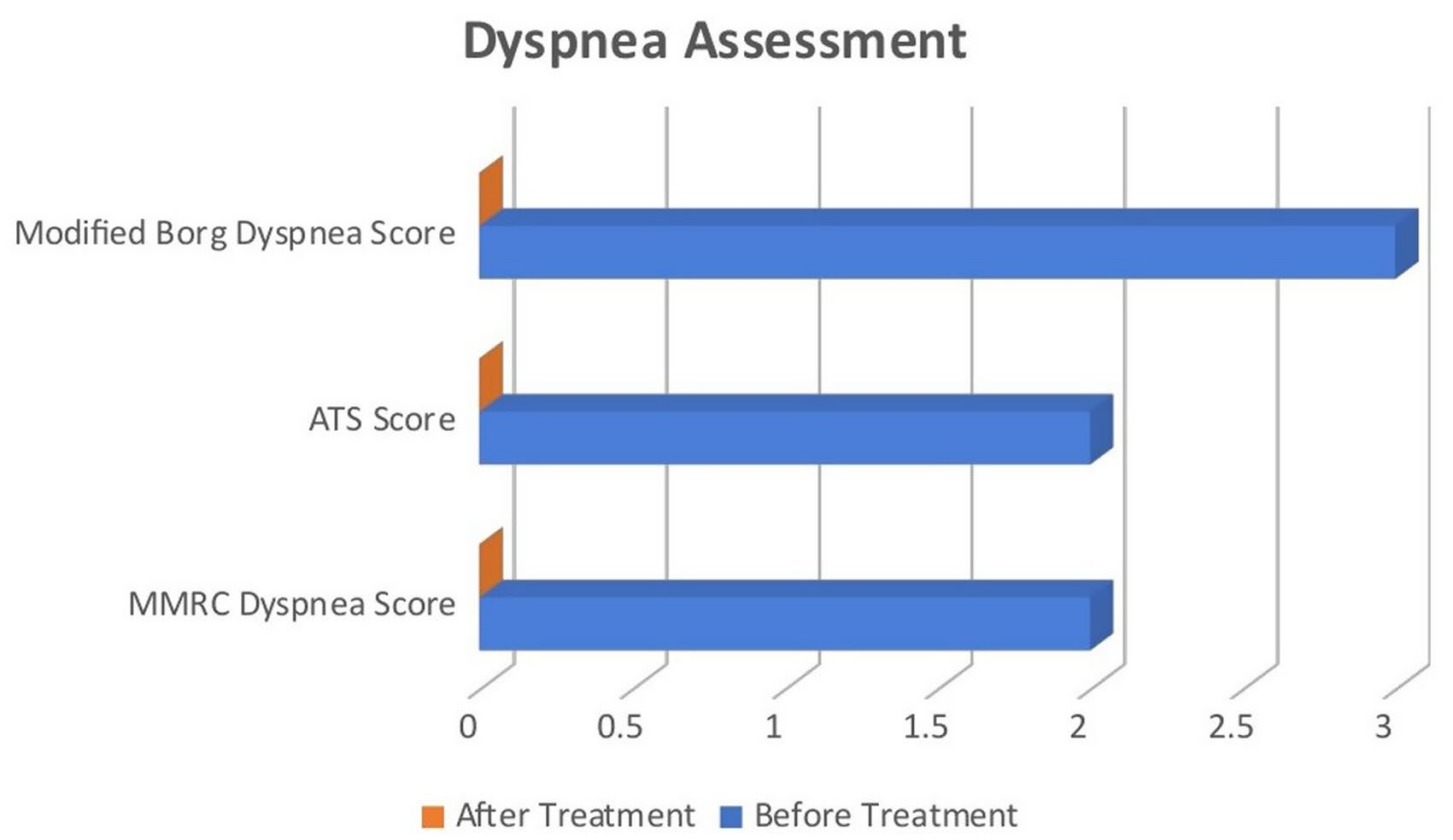
Assessment of dyspnea.

The patient was monitored throughout treatment, both clinically and radiologically. Clinicians and the patient noted improvement during the treatment period. Chest pain improved (VAS 8/10 to 1, Figure 2), dyspnoea resolved (mMRC grade 2 to 0; Modified Borg Scale 3 to 0, Figure 3), and cough, chest discomfort, irritability, and anorexia decreased (Table 1). Respiratory examination findings also improved, with better chest expansion, reduction in percussion dullness, and improved air entry into the left lung fields.

**Figure 4:**
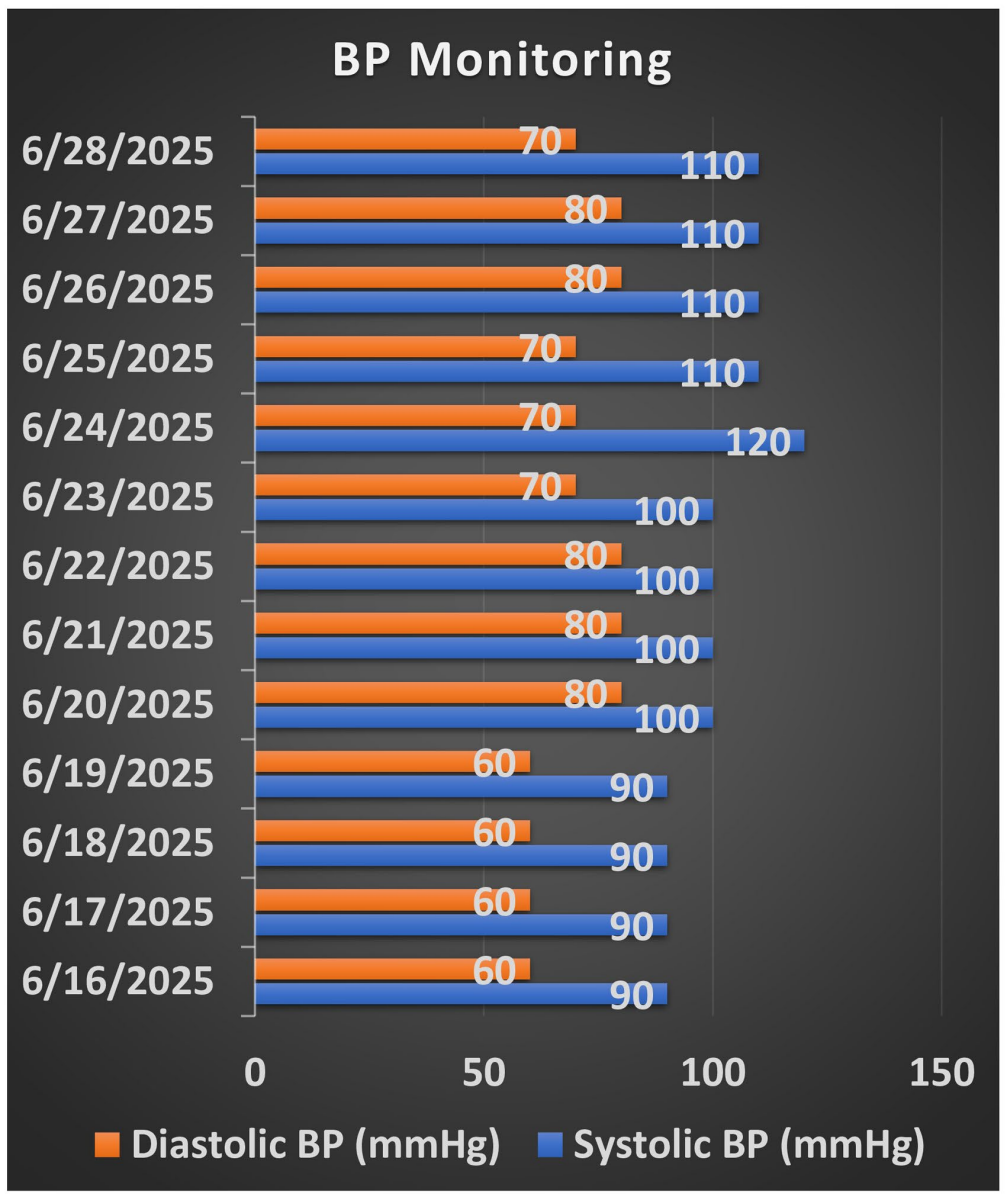
BP monitoring.

Blood pressure improved from 90/60 mmHg at presentation to 110/70 mmHg, reflecting haemodynamic stabilisation (Figure 4). Chest radiography after the completion of treatment demonstrated near-complete resolution of the left-sided pleural effusion (Appendix A), correlating with clinical recovery. The patient demonstrated good adherence to the Ayurvedic treatment, with no reported tolerability issues. No adverse events or deterioration were observed during treatment, and the patient remained clinically stable with no recurrence of symptoms and a normal X-ray at a 1-year follow-up visit, indicating a favourable outcome.

### Patient perspective

I initially experienced severe chest pain and difficulty breathing, which made routine activities difficult. I was advised to undergo a procedure (pleural tapping), but I was reluctant to undergo an invasive procedure. After discussing with my family, we decided to first try Ayurvedic treatment as a non-invasive option before considering any procedure.

After starting Ayurvedic medicines, I began to feel relief from my symptoms within a few days, particularly with regard to chest pain and breathlessness. By the end of 12 days, I felt much better and was relieved that no invasive procedure was required. I am satisfied with the treatment and the overall improvement in my condition.

## Discussion

Pleural effusion is a major contributor to respiratory morbidity, with idiopathic causes accounting for a significant portion of the illness burden, particularly in settings with limited resources and poor socioeconomic status. Even if the hazards, invasiveness, and cost of procedures such as thoracentesis limit their therapeutic efficacy, these concerns often restrict their use in resource-constrained communities.

These limitations highlight the need to evaluate conservative, cost-effective therapeutic strategies that may lead to clinical and radiological improvement while reducing procedural dependence, as illustrated by the current case, which used a non-invasive Ayurvedic intervention under close clinical monitoring (6, 7).

Elevated inflammatory markers are closely associated with pleural inflammatory activity and disease resolution. In this instance, attenuation of systemic inflammation was indicated by significant decreases in ESR (86 to 18 mm/h) and platelet count (6.79 to 2.74 × 10^1^/μL), which coincided with clinical and radiological improvement (Table 1). However, CRP was not measured, limiting the direct assessment of inflammatory activity. While the increase in haemoglobin and reductions in ESR and platelet count were consistent with the resolution of inflammation, these findings were temporally associated with the intervention. (8–11)

The diagnosis of unilateral presumed inflammatory/idiopathic pleural effusion was supported by the clinical and radiological exclusion of cancer, heart failure, hepatic, and renal aetiologies.

From an integrative perspective, the Ayurvedic formulation used in this case was selected based on classical descriptions of Uraḥstoya (12), conditions characterised by pathological fluid accumulation and impaired tissue metabolism. The Ayurvedic formulations and their classical rationale, alongside proposed biomedical mechanisms, are summarised in Table 4.

**Table 4:** Proposed biomedical mechanisms (derived from classical rationale, preclinical, experimental, and limited clinical evidence).

Drug/formulation	Mechanisms of action of Ayurvedic interventions	Proposed biomedical mechanisms *(based on classical, preclinical, and limited clinical evidence)*
Lakshadi Guggulu	Laksha (*Kerria lacca* Kerr) is indicated in thoracic injury (Uraḥkṣatahara) (16) and hemostatic regulation (Shonitasthapana) (17); Guggulu shows Lekhana (reducing) and Shothahara (anti-inflammatory) actions, which may support pleural fluid resolution (18)	*Commiphora mukul* (Hook. ex Stocks) Engl.-guggulsterones may modulate NF-κB and COX pathways → suggesting anti-inflammatory and pro-resolution effects (primarily based on clinical evidence) (19, 20)
Lakṣmīvilāsa Rasa	Kapha-Vatahara; Ushna virya (thermogenic) promotes Kapha liquefaction, relieves Srotorodha (airway obstruction), and exerts a Kledahara (fluid-reducing) effect in Uraḥstoya (21)	Polyherbo-mineral formulation may possess anti-inflammatory and immunomodulatory effects, and their potential roles in mucolysis and fluid regulation are hypothesised, with limited clinical evidence (21).
Yashtimadhu Churna (*Glycyrrhiza glabra* L.)	Included in 11 Mahakashaya out of them, Vamanopaga and Kanthya categories, indicating regulatory effects on Udana Vayu (upper respiratory functional axis) (22). Its Guru–Snigdha (demulcent, lubricating), Sheeta virya (cooling), Trishnaghna (thirst-reducing) properties may support mucosal protection and fluid balance (23–26)	Glycyrrhizin has been reported to possess demulcent, anti-inflammatory, cytokine-modulating, and mild expectorant actions. (supported mainly by preclinical and limited clinical evidence) (27–29)
Talisadi Churna	Indicated in Kasa–Shwasa, Parshwashoolahara, and Kasa-Shwasahara; Deepana, Pachana–Lekhana may help reduce Ama and Kapha-mediated fluid accumulation (30)	Piperine, gingerols, and cinnamaldehyde may exhibit anti-inflammatory, antitussive, antihistaminic, and airway-modulating effects. (Clinical study) (31)
Ashwagandha Churna (*Withania somnifera* (L.) Dunal)	Balya–Rasayana; Tikta–Kashaya rasa with Ushna virya may promote Soshana (fluid absorption) and Shothahara action (32)	Experimental studies suggest that withaferin A may modulate inflammatory pathways, including NLRP3 and TGF-β signalling (33).
Amalaki Churna (*Phyllanthus emblica* L.)	Rasayana; Tridoshaghna; Ruksha–Kashaya properties may contribute to Shoshana (fluid resorption) and Sothahara effects (34)	Vitamin C and polyphenols are known to have antioxidant, anti-inflammatory, antitussive, and mucoregulatory effects. (preclinical evidence) (35)

Serial chest radiographs demonstrate marked regression of the left-sided pleural effusion, accompanied by improvement in pleuritic pain and dyspnoea. However, spontaneous resolution of an underlying inflammatory pleural effusion remains a plausible alternative explanation for the observed improvement. In the context of negative microbiological findings, radiological clearance accompanied clinical improvement. Similar conservative and integrative approaches to pleural effusion have been reported in the literature. Ma et al. described a case of undiagnosed pleural effusion managed with Traditional Chinese Medicine, while Wankhade et al. reported adjunctive individualised homoeopathic management in a patient with tubercular pleural effusion. Mobolaji and Omole described successful non-operative management of pleural effusion using a collaborative conservative approach (13–15).

Compared with previously published reports, the present case documents clinical improvement accompanied by near-complete radiological resolution of presumed inflammatory/idiopathic pleural effusion following an exclusively non-invasive Ayurvedic approach. The report further contributes detailed clinicoradiological follow-up and structured outcome assessment, adding to the limited literature on conservative management of pleural effusion.

The strengths of this case include the successful use of an exclusively Ayurvedic treatment approach, without the use of modern medicine or pleural tapping. Additional strengths included detailed clinicoradiological monitoring, the use of standard symptom scales, and documentation of improvement through imaging and laboratory parameters. The non-invasive, cost-effective approach, with its short treatment duration and early recovery, adds to its clinical relevance, particularly in resource-limited settings (Table 5).

**Table 5:** Timeline of events.

Time point	Clinical event
10–15 days before presentation	Onset of pleuritic chest pain, dry cough, anorexia, irritability, and weight loss
2 days before presentation	Fever with chills; treated symptomatically with antipyretics
Day 0 (Presentation)	Presented with dyspnoea (mMRC grade 2) and severe pleuritic chest pain (VAS 8/10)
Day 1–2	Diagnostic evaluation performed: Chest X-ray and ultrasonography confirmed moderate left-sided pleural effusion; sputum AFB smear and CBNAAT negative
Day 2	Presumed inflammatory/idiopathic pleural effusion diagnosed based on clinical, radiological, and laboratory assessment
Days 2–13	Conservative Ayurvedic treatment was initiated and continued under close clinical monitoring.
Day 7	Clinical improvement was observed with a reduction in chest pain, dyspnoea, cough, and chest discomfort.
Days 12–13	Repeat chest X-ray demonstrated near-complete resolution of pleural effusion; symptoms markedly improved; and thoracocentesis not required.
1-month follow-up	Patient remained clinically stable with no recurrence of symptoms.
1 year follow up	No recurrance

This case is limited by its single-patient design, lack of pleural biochemical analysis, and absence of CRP testing. The absence of pleural fluid analysis limited the classification of the effusion and reduced diagnostic certainty. Additionally, in the absence of CRP and pleural fluid markers, a reduction in inflammation was inferred from the decline in ESR and clinical improvement, rather than being directly confirmed, which may affect internal validity and causal interpretation. Although microbiological tests were negative, the complete exclusion of tuberculosis remains a challenge. Furthermore, spontaneous resolution of certain inflammatory or parapneumonic pleural effusions cannot be ruled out. Therefore, the findings should be interpreted cautiously and considered hypothesis-generating, requiring further studies.

## Conclusion

This case describes a clinical observation of improvement in a patient with *presumed inflammatory/idiopathic pleural effusion* managed with a safe, conservative, non-invasive, and cost-effective Ayurvedic approach. Although clinical and radiological improvements were observed, further well-designed, multicentre pragmatic studies incorporating standardised diagnostic evaluation, pleural fluid analysis, inflammatory biomarkers, and long-term follow-up are required to assess the reproducibility of these observations and explore potential causal relationships.

## Data Availability

The original contributions presented in the study are included in the article/Supplementary material; further inquiries can be directed to the corresponding author.
