# Supplementary figures and images for: A clinico-radiological evidence of Ayurvedic management for presumed inflammatory/idiopathic pleural effusion: case report
Source: Front Med (Lausanne). 2026 Jul 14;13:1803298. doi: 10.3389/fmed.2026.1803298 (PMC13366123; doi:10.3389/fmed.2026.1803298)

**Appendix A. Radiological Assessment**


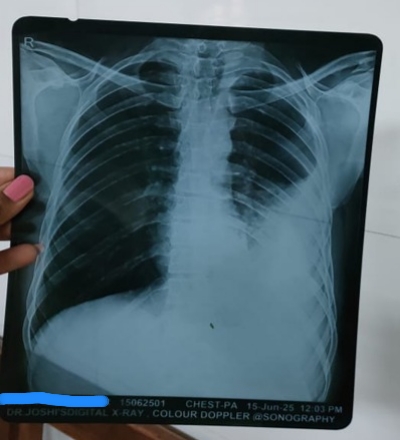

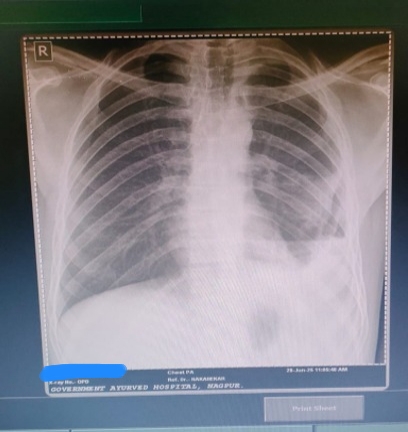


**Before Treatment 15/06/25**

**After Treatment 28/06/25**


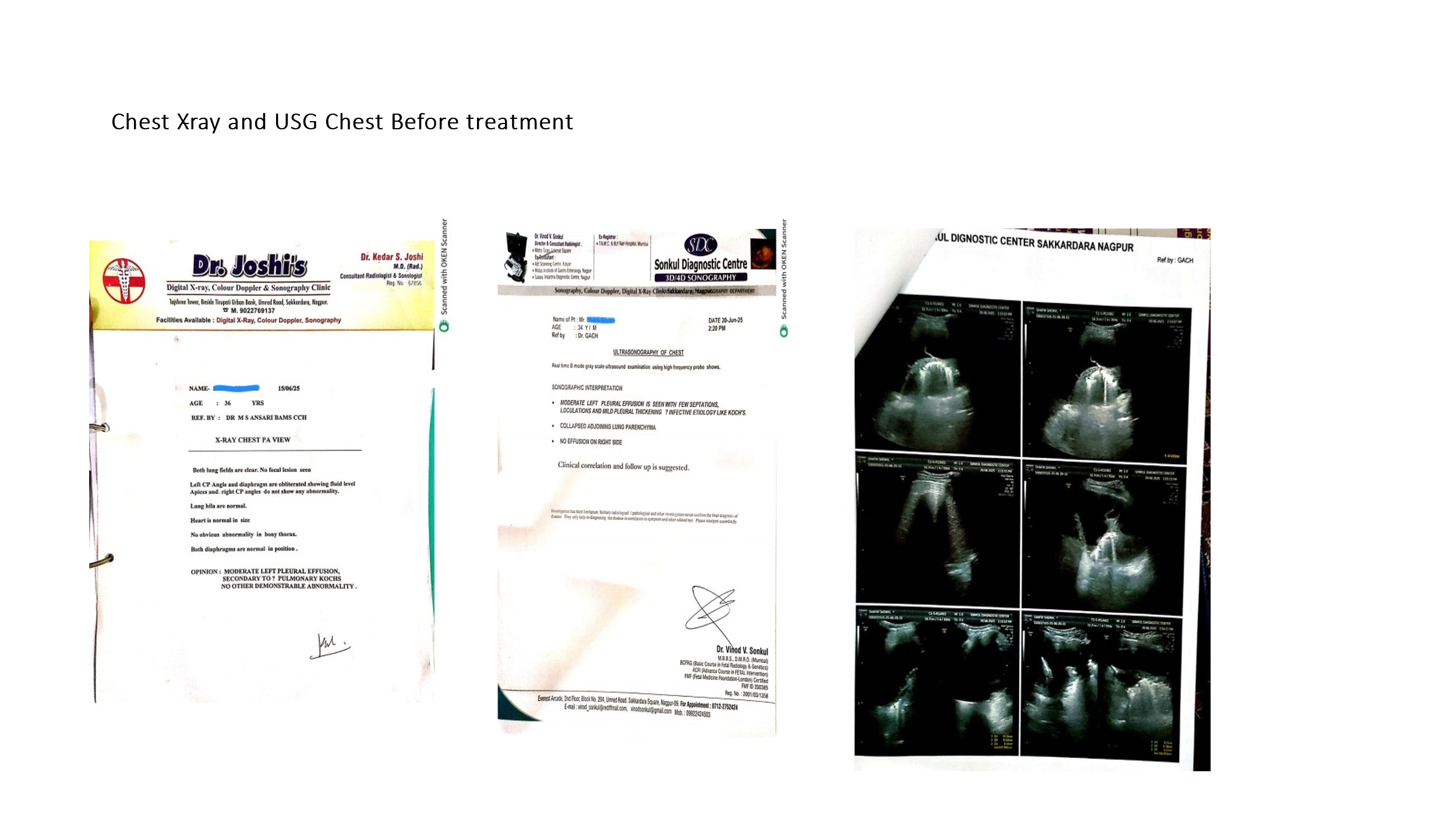


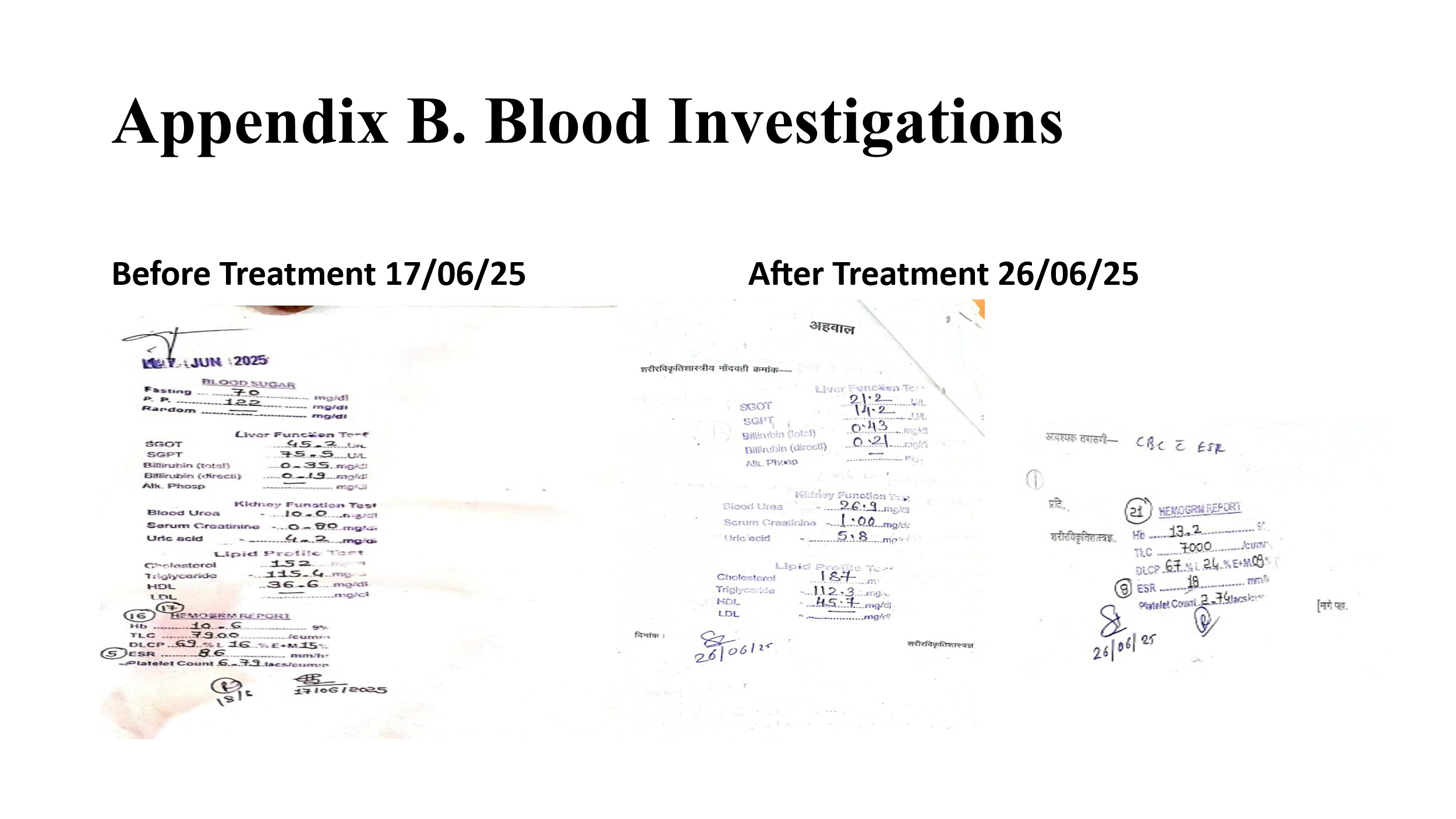


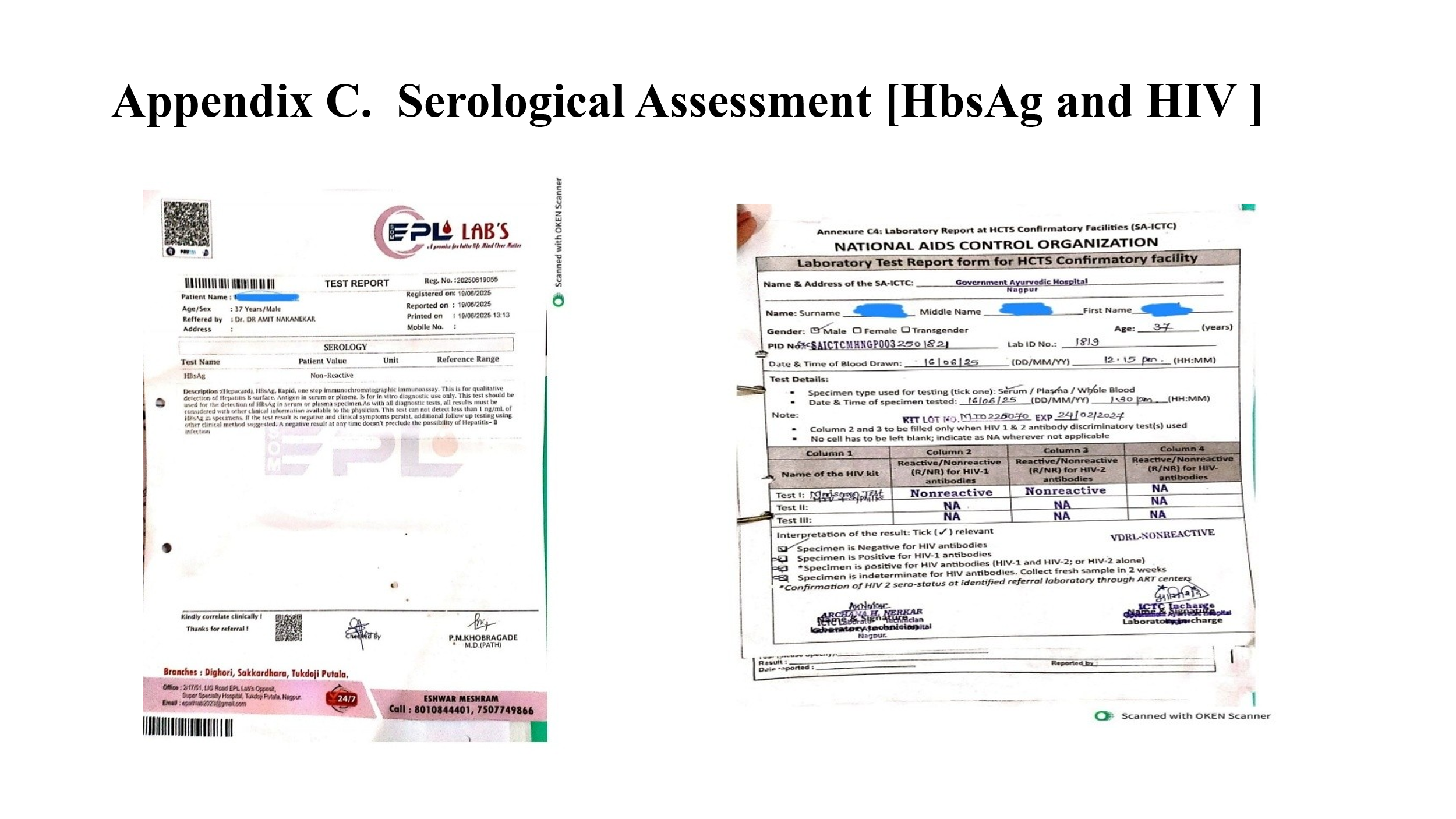


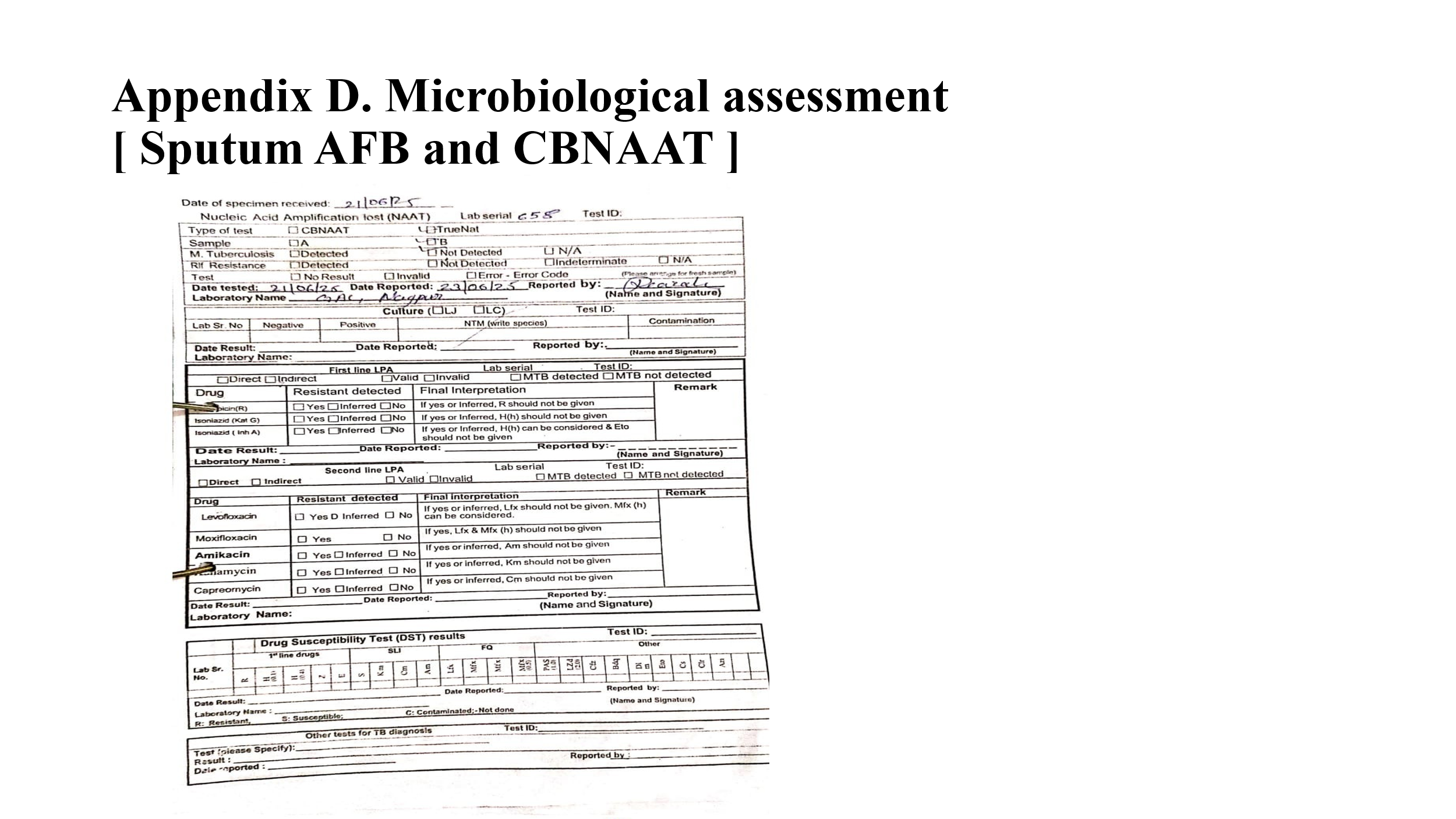


**Appendix E. [ ECG] (17/06/25)**

**
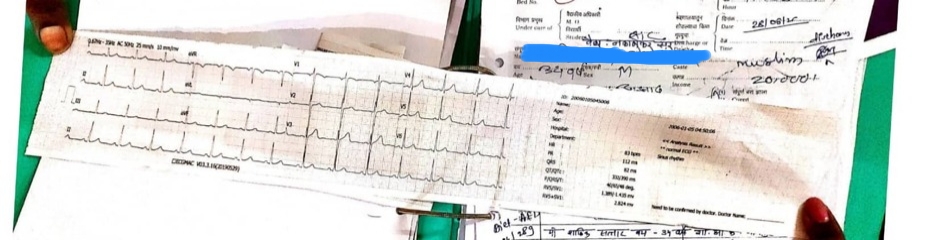
**

Supplement: Supplementary file 1 [file Supplementary_file_1.docx]
